# The Medical Council

**Published:** 1899-06-10

**Authors:** 


### THE MEDICAL COUNCIL.

The sixty-seventh session of the General Medical Council was opened on May 22nd with the customary address from the president, Sir William Turner, who referred to the loss which the Council had sustained by the decease of the late Sir William Roberts, and welcomed as his successor, in the representation of the University of London, Dr. Philip Henry Pye-Smith, F.R.S., who was introduced and took his place at the Council table. The President gave a brief summary of the principal business likely to be brought under consideration, and a history of negotiations in which he had been engaged with various departments of the Government. The customary returns of the results of examinations for qualifications, and of competitive examinations for the public services, were received from the several authorities concerned, and votes of thanks to these authorities were passed in the usual way. Attention was called to the fact that out of 36 candidates for commissions in the Royal Army Medical Corps, and 24 for commissions in the medical department of the Royal Navy, no less than 35 of the former and 22 of the latter were passed by the examiners as qualified for vacancies. A motion by Mr. Brown, with reference to the different proportion of passes to rejections in two different examinations, was disposed of by the "previous question," and the same fate, by a majority of 23 votes to four, befel a motion in which Mr. Horsley attempted to censure the President with regard to one of his rulings from the chair in the preceding session of the Council. The President first showed that the circumstances were not accurately stated in Mr. Horsley's notice. A new standing order was passed, on the recommendation of the Executive Committee, with regard to the conditions under which documents belonging to the Council should be made available for examination by individual members; and the Council adjourned at four o'clock in order to liberate the members for attendance upon various committees.

The first business of Wednesday, the 23rd, was the consideration of a motion by Mr. Brown, seconded by Mr. Horsley, "That in the opinion of the Council the time has come when it is expedient to confer on the registered medical practitioners resident in England and Wales the power of returning an additional member to the General Council, as provided for by the Medical Act of 1886." The Council had previously received from the Council of the British Medical Association a resolution passed by that body, in which it was said to be "felt" that the "general practitioners in the United Kingdom are entitled to demand a much larger share of direct representation on the General Medical Council." Mr. Brown introduced his motion in a very temperate speech, but was unable to adduce any arguments in its favour other than numerical ones; and it was shown by many other speakers that these formed but a subordinate element in the case. Ultimately the motion was lost by 21 votes to 7.

One of the penal cases coming before the Council, in which a practitioner was charged with assisting one Rowland to obtain registration in the Medical Register by personating a qualified man named Nugent, led to the appointment of a committee to report on the question of personation generally. Various other matters were referred to committees for report, or were otherwise advanced a stage.

On Thursday, Friday, and during most of the sitting on Saturday the Council was engaged with disciplinary questions. Samuel Bingham Shekleton was removed from the Register for having falsely certified that the man Rowland, referred to above, was Edward Joseph Nugent; and it was directed that the documents referring to the case should be brought to the attention of the proper authorities in New South Wales, where the real Nugent is said to be practising. James Jerome McKay was removed from the Register for having given false evidence at a coroner's inquest, to the effect that he had made a complete post-mortem examination in a case in which it was afterwards proved that he had not done so, and that his evidence as to the cause of death was untrue. A case of much interest and importance was that of Dr. William Stewart, who was charged with "covering" an unqualified person named Burgess. It appeared from the evidence that Dr. Stewart is medical officer to a so-called provident dispensary, and that Burgess is employed, nominally as a dispenser, not by Dr. Stewart, but by the "committee" of this dispensary; and on this ground Dr. Stewart endeavoured to disclaim all responsibility for Burgess's acts. After a very long and patient hearing, the Council decided that the charge against Dr. Stewart was proved, but postponed judgment for six months, in order to afford him an opportunity of reconsidering his position. Neville Holland was removed from the Register for the second time (having only lately been restored), for covering an unqualified person named Blumenthal; and William Henry Cossens was removed in consequence of having been convicted of a misdemeanour. The greater part of Saturday's sitting was occupied in considering *in camerâ* certain findings of fact by the Dental Committee in relation to a charge of covering an unqualified person which was brought against Mr. Clement Maguire Kershaw; and, in the event, the matter was adjourned until November, so that Mr. Kershaw, like Dr. Stewart, might have an opportunity of "considering his position."

Monday was occupied in discussing the reports of three committees, and the action to be taken on their recommendations. The Education Committee presented an interim report on the proposal to raise the minimum requirements of the Council as regards preliminary examinations. In view of the answers received from the various educational authorities the committee recommended that the Council should extend the period of reference to them, and should provide them with the means of obtaining expert assistance. Sir J. Batty Tuke, chairman of the committee, expressed a hope that if this were done they would be able by November next to present a scheme for the gradual raising of the junior examinations which would meet with general acceptance. The recommendation was adopted. The report from the Midwives' Bill Conference Committee on the Midwives' Bill, 1899, did not, however, meet with so unanimous a reception. After stating certain objections to clauses in the Bill the committee recommended that the Council inform the Lord President of the Privy Council that they were unable to approve the Bill unless it were recast in accordance with certain suggestions contained in the report. Now, among other matters, the committee had expressed the opinion that all the members of the Central Midwives Board should be registered medical practitioners, and it seemed to Sir R. Thorne Thorne to be wrong that they should attempt to get the proposed board entirely composed of members of their profession, seeing that if the Bill passed they would have no right of intervention in every case of labour because the majority of births were natural. He therefore, although chairman of the committee, declined to move the adoption of the report, and proposed an amendment to the effect that the Council should withhold its approval from the paragraph in question. The report, as amended in accordance with Sir R. Thorne Thorne's suggestion, was adopted.

The report of the Medical Aid Associations Committee included a recommendation in favour of the formation of a board of conciliation consisting of representatives of the friendly societies and the medical profession to decide the question at issue. Mr. Victor Horsley, however, thought that such a recommendation almost took the Council beyond its province, and Sir R. Thorne Thorne thought it was a matter with which the Council should have nothing to do. In the end the recommendation was carried, but only after being whittled down to the level of a pious opinion by the addition of the very important proviso that it be understood that it is inexpedient that the Council should take any part officially in the formation of the board.

On Tuesday the discussion on medical aid associations was continued from a somewhat different point of view, and ultimately a resolution was adopted declaring that the Council strongly disapprove of medical practitioners associating themselves with medical aid associations, by which systematic canvassing and advertising for the purpose of procuring patients are practised.

